# The female effect—how female receptivity influences faecal testosterone metabolite levels, socio-positive behaviour and vocalization in male Southern white rhinoceroses

**DOI:** 10.1093/conphys/coab026

**Published:** 2021-04-28

**Authors:** Julia Jenikejew, Jella Wauters, Martin Dehnhard, Marina Scheumann

**Affiliations:** 1 Institute of Zoology, University of Veterinary Medicine Hannover, Bünteweg 17, 30559 Hannover, Germany; 2 Department of Reproduction Biology, Leibniz Institute for Zoo and Wildlife Research, Alfred-Kowalke-Straße 17, 10315 Berlin, Germany

**Keywords:** *Ceratotherium simum*, zoo, faecal androgens, oestrus, sociosexual behaviour, non-invasive monitoring

## Abstract

Testosterone is known to be essential for sexual maturation as well as for the display of behavioural traits linked to reproduction. At the same time, external factors such as the presence of receptive females may affect testosterone levels, stressing the hormone’s substantial role in reproductive success. It is therefore of major interest to investigate the links between androgens, behaviour and the social environment especially in species that rely on a resilient reproduction rate, such as the white rhinoceros (WR). We collected faecal samples of 16 male Southern WR (*Ceratotherium simum simum*) aged between 1 and 44 years from 11 European zoos. Audio and video recordings were simultaneously taken from five of the study males that were sexually mature and had direct contact with receptive females. Our results showed a positive correlation of faecal testosterone metabolite (fTM) concentrations and progressing age up until adulthood followed by a decline in older males. While previous reproductive success did not show any effect, the access to receptive females resulted in higher fTM levels. Thereby, fTM concentrations remained at the same level regardless of the receptivity phase, while social cohesion with respective females, affiliative behaviour as well as call rates of Pant and Hiss distinctly peaked during the receptive compared to the non-receptive periods. Conclusively, the immediate presence of receptive females poses a female effect that enhances the overall androgen levels in males and, thus, might facilitate their reproductive success. However, androgens do not seem to be the main driver of behavioural changes during courtship or mating. By linking endocrinological and socio-behavioural factors, we were able to provide an applicable basis for non-invasive monitoring of reproductive behaviour in male WR in captivity, thereby contributing to deeper understanding of potential reproduction impairments in a species whose population in captivity remains not fully self-sustaining.

## Teaser

Faecal testosterone levels were compared among 16 male white rhinoceroses (WR). Presence of receptive females was positively correlated with elevated fTM levels in adult males. While socio-positive behaviour and vocalization peaked during a female’s oestrus, fTM levels remained unchanged. The findings highlight male indicators of female receptivity in captive WR.

## Introduction

Over the past decades, research studies have comprehensively identified testosterone (T) as essential for the development of primary and secondary male sexual characteristics (Heldmaier *et al.*, 2013) and as a central mediator of various behavioural traits associated with reproduction, for instance sexual aggression (Wingfield *et al.*, 1990), courtship (Adkins-Regan, 1998) and mating behaviour (Lincoln *et al.*, 1972). Withal, studies in various mammalian species could show that levels of androgens in males can be affected by external factors, such as the presence of females (e.g. Rose *et al.*, 1972; McDonnell and Murray, 1995; Muller and Wrangham, 2004; Hirschenhauser and Oliveira, 2006) as well as the degree of association with them (Dloniak *et al.*, 2006), therefore emphasizing a female effect on male reproductive state. Moreover, T levels were proved to correlate with reproductive-related behaviour as well as with vocalization. During the mating season, high rates of aggressive behaviour were reportedly associated with a rise in T levels (e.g. Jainudeen *et al.*, 1972; Poole and Moss, 1981; Lincoln and Ratnasooriya, 1996; Cavigelli and Pereira, 2000; Muller and Wrangham, 2004; Gould and Ziegler, 2007), while further studies could show that social dominance positively correlates with T concentrations as well (e.g. Beehner *et al.*, 2006; Muehlenbein and Watts, 2010; Wang *et al.*, 2012). Vocal behaviour has been demonstrably linked to T levels in males too, proving that acoustic parameters change depending on the sender’s androgen state (e.g. Floody *et al.*, 1979; Galeotti *et al.*, 1997; Higham *et al.*, 2013). In various mammalian species, higher T levels have been significantly associated with higher pitched calls (Barelli *et al.*, 2013; Fedurek *et al.*, 2013, 2016), increased call duration (Charlton *et al.*, 2011) as well as increased call rates (Zimmermann, 1996; Pasch *et al.*, 2011). Moreover, playback experiments in brown mice (Pasch *et al.*, 2011) and giant pandas (Charlton *et al.*, 2012) demonstrated that females were able to identify vocalizations of males with higher T values and reacted with a greater response. Hence, while it has been established that T plays a substantial role in reproduction by enhancing the reproductive success (e.g. Mills *et al.*, 2009; Negro *et al.*, 2010), it has received rather marginal attention in a species that strongly relies on a sustained improvement of its reproductive rate in captivity—the white rhinoceros (*Ceratotherium simum*, WR).

As the WR population in the wild continues to be threatened by heavy poaching and persistent droughts (Ferreira *et al.*, 2019; Emslie *et al.*, 2020; Emslie, 2021), *ex situ* breeding is a crucial backbone, serving as both a genetic and demographic reservoir for reintroduction and population strengthening (Seror *et al.*, 2002; Swaisgood *et al.*, 2006). Despite long-standing research on reproduction and husbandry as well as intensive breeding efforts, the European WR population in captivity still experiences low reproductive rates and, hence, remains not self-sustaining (Hermes *et al.*, 2004; Swaisgood *et al.*, 2006; van der Goot, 2015; Versteege, 2018). Researchers investigating the reasons for this instability point to a variety of physiological and external factors, including abnormalities of the endocrine cycle (e.g. Schwarzenberger *et al.*, 1998; Patton *et al.*, 1999; Schwarzenberger *et al.*, 2003), reproductive pathologies (e.g. Hermes *et al.*, 2006) as well as deficiencies in socio-sexual behaviour based on unfavourable group composition (e.g. Seror *et al.*, 2002; Metrione *et al.*, 2007). While the vast majority of endocrinological studies in WR investigated progesterone levels and its development and reproductive function in females (e.g. Radcliffe *et al.*, 1997; Schwarzenberger *et al.*, 1998; Patton *et al.*, 1999; Brown *et al.*, 2001; Hermes *et al.*, 2006; Sós *et al.*, 2006; van der Goot *et al.*, 2013; van der Goot *et al.*, 2015b; Hermes *et al.*, 2020; Pennington *et al.*, 2020), only a few studies examined androgen concentrations in males, focusing on environmenta and social effects (Brown *et al.*, 2001; Kretzschmar *et al.*, 2004; Christensen *et al.*, 2009), territorial behaviour (Rachlow *et al.*, 1998) as well as breeding success (Ververs, 2018; Kretzschmar *et al.*, 2020). Whereas the authors describe an increase in faecal androgen metabolite levels during sexual maturation (Kretzschmar *et al.*, 2004; Ververs, 2018) and agree on the importance of T for the reproductive state, there are some discrepancies in their results regarding the effect of females on the androgen metabolite values. While Ververs (2018) did not find any significant differences in faecal androgen levels between adult bulls kept with females and the ones that were isolated, Christensen *et al.* (2009) and Kretzschmar *et al.* (2004) could both determine a female effect, with solitary bulls having significantly lower androgen levels than those in close proximity to the other sex. However, Ververs (2018) and Christensen *et al.* (2009) both compared different males varying in their husbandry conditions and did not account for the reproductive state of the females. Kretzschmar *et al.* (2004) on the other hand identified the receptive phase of females by considering a male closely accompanying the female as a behavioural indicator of oestrus. They subsequently compared individual faecal testosterone metabolite (fTM) concentrations during the female’s presence with fTM concentrations during solitary periods but did not compare males in different oestrous periods of the females among each other. Hence, the question of whether it is the merepresence of females or their actual oestrus phase that potentially affects the androgen levels in WR bulls remains considerably open. Furthermore, the majority of previous studies focusing on androgen concentrations in male WR were conducted in wild conditions (Rachlow *et al.*, 1998; Kretzschmar *et al.*, 2004, 2020) or at least semi-wild (Ververs, 2018) and only a few in captivity (Brown *et al.*, 2001; Christensen *et al.*, 2009). Considering that individuals of the same species can substantially differ in physiological (e.g. Naidenko *et al.*, 2011), morphological (e.g. Gidna *et al.*, 2013), cognitive (e.g. Benson-Amram *et al.*, 2013) as well as behavioural (e.g. Stoinski *et al.*, 2003; Hosey, 2005) parameters between wild and captive conditions, further investigations of captive animals are required. This is of particular importance in WR, as reproductive impairments in this species are more severe in *ex situ* cases (Roth, 2006; Swaisgood *et al.*, 2006).

The behavioural repertoire of WR has been investigated in a number of previous studies (e.g. Owen-Smith, 1973, 1975). Some of the described behavioural patterns of males proved to be reliable indicators of a female’s receptivity (Owen-Smith, 1975; Penny, 1987). Closely following and guarding a female as well as placing their head on the female‘s rear and repeated mounting attempts have been established as clear predictors of impending copulation. In addition to a highly pronounced olfactory communication, including marking with urine and ritualized dung kicking as well as sniffing and flehming that are essential for the males to establish their territories and locate females (Marneweck *et al.*, 2017, 2018), previous studies identified vocal communication as relevant for reproduction as well. Especially the Pant call was repeatedly described as a cohesive call type, serving a directed communication and mainly occurring during affiliative interactions or social exploration (Owen-Smith, 1973; Policht *et al.*, 2008). Besides, a conspicuous variation of the Pant call, also known as Hic, has been described as specifically characteristic during courtship, particularly for free-ranging bulls (Cinková and Policht, 2016; Cinková and Shrader, 2020). A recently described sex difference in Pant call rates additionally indicates a socio-sexual function of this call type, with adult bulls panting significantly more often than adult females (Jenikejew *et al.*, 2020). A similar sex-specific variation in call rates has been also identified for the Hiss (Jenikejew *et al.*, 2020), a call type associated with agonistic interactions (Owen-Smith, 1973; Policht *et al.*, 2008), with females calling significantly more often than males.

However, to date, there are no findings on the relation between the occurrences of behaviour or call types that are relevant for reproduction and the hormonal state in WR males. Moreover, there is no information on potential effects of female’s oestrus on these different parameters and how they might develop over the course of the receptive period. Relevant findings could provide information on the function of testosterone for the display of behaviour essential for reproduction. In the further course, this information might also be implemented in breeding management, as the absence of or aberration from said indicators could provide insights into the impairment of reproductive success in WR.

Accordingly, the aim of the present study was to investigate hormonal as well as behavioural and acoustic factors that are relevant for reproduction in captive male WR and to provide a comprehensive insight by establishing their interrelationship. In order to do so, we first compared mean fTM levels between age classes and breeding activity to assess how androgen status change depending on sexual maturation as well as previous reproductive success. Subsequently, we examined the effect of receptive females on mean fTM levels in sexually mature bulls. Finally, we tested whether the females’ receptive and non-receptive periods could predict fTM levels, the social cohesion between males and respective females as well as behavioural rates (affiliative, agonistic and olfactory) and call rates of Pant and Hiss.

## Methods

### Ethical statement

The article contains behavioural and hormonal data on zoo animals derived from non-invasive collection of faecal samples and recordings. No animal was taken out of its usual environment or invasively manipulated by the authors. The authors received permission to record behavioural data and collect faecal samples on the ground of the respective zoo.

### Study sites and animals

The study involved 16 male Southern WR (*Ceratotherium simum simum*, SWR) at 11 European zoological institutions. With the exception of two young males being kept separately (see Planète Sauvage 2 in Table 1), all groups consisted of one adult male, one to four adult females and in three zoos there were additional juvenile and subadult females and males present. In nine groups, all animals had direct contact to each other during the day, while in two groups adult males were separated by a fence that only enabled limited (visual, acoustic and olfactory) contact (Dortmund and Amnéville). All study males were categorized according to their age at the time of data collection (0–3 years, juvenile; 3–5 years, subadult; 5–10 years, adolescent; ≥ 10 years, adult; Owen-Smith, 1973, 1988). Sexually mature males (≥ 5 years; Versteege, 2020) were additionally classified according to their reproductive success (existence of sired offspring) as well as the extent of contact with receptive females during data collection (direct contact: visual, acoustic, olfactory and tactile contact; no contact: visual, acoustic, olfactory but not tactile contact or no receptive females available; Table 1). Females’ receptivity was determined based on observations of sexual behaviour (head placing, mounting, copulation) directed towards them by males, as previous studies had established this behaviour as being a reliable indicator of oestrus (Patton *et al.*, 1999; Swaisgood *et al.*, 2006).

**Table 1:** Information on study males during observation period

ID	Zoo	Sampling year	Age (years)	Sexual maturation	Successfully sired before	Number of adult females in the group	Number of receptive females in the group	Contact to receptive female(s)
Floris	Osnabrück	2014	38	Yes	No	3	2	Direct contact
Bantu	Augsburg	2014	9	Yes	No	3	2	Direct contact
Amari	Dortmund	2014	9	Yes	Yes	3	0	No contact
Dino	Erfurt	2015	22	Yes	Yes	2	0	No contact
Martin	Hodenhagen	2015	22	Yes	Yes	5	1	Direct contact
Abasi	Hodenhagen	2015	4	No	No	5	1	Direct contact
Dinari*	Hodenhagen	2015	2	No	No	5	1	Direct contact
Lekuru	Gelsenkirchen	2015	11	Yes	No	2	0	No contact
Kimba	Schwerin	2018	10	Yes	No	2	2	Direct contact
Harry	Münster	2018	28	Yes	Yes	2	1	Direct contact
Amiri	Münster	2018	1	No	No	2	1	Direct contact
Benny**	Amnéville	2018	14	Yes	Yes	1(3)	0	No contact
Timbo	Amnéville	2018	1	No	No	3	0	Direct contact
Shaka	Knuthenborg	2019	16	Yes	No	2	0	No contact
Jambo	Planète Sauvage 1	2019	44	Yes	No	1	0	No contact
Goliath	Planète Sauvage 2	2019	7	Yes	No	0	0	No contact

^*^Study male was observed again in Planète Sauvage 2 but only data from Hodenhagen are included in this study.

^**^Study male was kept in direct contact with one female and with three other females in a neighbouring enclosure separated through a fence.

Direct contact: visual, acoustic, olfactory and tactile contact with receptive females; no contact: visual, acoustic, olfactory but no tactile contact with receptive females (separation through fence) or no receptive females available at all.

### Vocal and behavioural data collection

Over an average period of 18 consecutive observation days, simultaneous acoustic and behavioural recordings were taken of all individuals in the groups using focal animal sampling (Altmann, 1974). Each focal animal was observed for 10 min per session, resulting in 20 to 40 min daily observation time distributed between 8 am and 6 pm in a randomized order. In further analyses, only the recordings of the study males that were sexually mature and had direct contact with receptive females (*N*_DC_ = 5) were included. In doing so, 43 hours of data were analysed: 10 hours at Zoo Osnabrück (April 2014), 10 hours at Zoo Augsburg (July/August 2014), 3 hours at Serengeti-Park Hodenhagen (April/May2015), 10 hours at Zoo Schwerin (April/May 2018) and 10 hours at Zoo Münster (July/August 2018).

Video recordings were made using a digital camcorder (Sony DCR-SR36E, Sony Corporation, Tokyo, Japan). Audio recordings were made using a Sennheiser omni-directional microphone (Sennheiser MKH 8020, Sennheiser electronic GmbH & Co. KG, Wedemark-Wennebostel, Germany; flat frequency response from 10 to 20 000 Hz ± 5db) that was equipped with a wind shield and a boom pole. The microphone was connected to a digital recording device (Sound devices 702T State Recorder, Sound Devices LLC, Reedsburg, USA; frequency response: 10–40 000 Hz; settings: 44.1 kHz sampling rate, 16 Bit, uncompressed .wav format).

### Vocal and behavioural analysis

For behavioural coding, video recordings were synchronized with respective audio recordings and analysed using the ‘Observer XT’ software (version 12, Noldus Information Technology, Netherlands; Noldus, 1991). The analysis was conducted by two different observers (BC: Osnabrück, Hodenhagen, Schwerin; JJ: Augsburg, Münster). The Cohen’s Kappa coefficient was determined among the observers by comparing 15 pilot observations (total of 100 min). All $ {\includegraphics{\bwartpath coab026fx1}}$ values were ≥0.95, indicating a high interrater reliability (Landis and Koch, 1977).

Throughout the recordings, vocalizations were detected by auditory identification and categorized according to the literature (Owen-Smith, 1973; Policht *et al.*, 2008; Linn *et al.*, 2018). For each vocalization, the respective call type, sender as well as potential receiver were noted (see Jenikejew *et al.*, 2020 for detailed description). Vocalizations that due to ambient noises could not be reliably assigned to a call type or a sender were excluded from the analysis. A previous study (Jenikejew *et al.*, 2020) on vocal communication structure in SWR had demonstrated sex-specific differences in call rates for the contact call Pant (including its call variation Hic) as well as the agonistic call Hiss, emphasizing a relevant role of these two call types for mating behaviour. A further analysis in the present study therefore only included Pant and Hiss calls. Thereby, Pant calls were defined as bouts of repetitive calls produced during inhalation and exhalation (Policht *et al.*, 2008) and were mainly uttered when the male approached females, usually aiming to initiate sexual behaviour with one of them. Hiss calls were broadband noisy nasal sounds occurring single or in bouts (Policht *et al.*, 2008) that were uttered in aggressive contexts in order to displace or threat the potential receiver.

Each behaviour was coded considering proximity measurements of the focal male to present group members, taking adult body length (2.5–3 metres; Owen-Smith, 1973) as the measuring unit. The duration each focal male spent in close proximity (≤1 body length) to each group member was noted.

For each focal male the occurrence of affiliative, aggressive and defensive interactions and the respective interaction partner as well as olfactory behaviour were noted (see ethogram in Table 2). Affiliative interactions included social exploration of the interaction partner as well as socio-positive and sexual behaviours. Aggressive interactions were coded when the focal male displaced, attacked, chased, pushed or clashed horns, etc. with the interaction partner, whereas defensive interactions were coded when the focal male avoided or escaped from the interaction partner. Olfactory behaviour comprised marking as well as sniffing and flehming.

**Table 2:** Ethogram of olfactory, affiliative and agonistic behaviour of captive SWR

	Behaviour	Description
Olfactory	Marking	Focal animal urinates intermittently or spreads its defecation with its hind legs
	Sniffing	Focal animal explores ground/objects or urine/faeces by inclining towards it, ‘sliding’ along the surface with the snout
	Flehming	Focal animal opens its mouth and curls back its upper lip exposing its upper gum while inhaling
Affiliative	Following	Focal animal moves after a conspecific while it changes the location
	Snout contact	Focal animal explores the body of another conspecific (except the snout) with its snout
	Social Flehming	Focal animal flehms while scenting a defecating/urinating conspecific close by
	Naso-nasal sniffing	Focal animal contacts the nasal region of another conspecific with its own snout
	Ano-genital sniffing	Focal animal contacts the ano-genital region of another conspecific with its own snout
	Head placing	Focal animal lays its head on the back of another conspecific
	Body contact	Focal animal touches or brushes another conspecific while moving with any part of its body (except snout) or rubs itself against a conspecific
	Mounting	Focal animal climbs with its forelegs on another conspecific
	Copulation	The animals mate: the bull inserts his penis into the cow
Aggressive	Displace	Focal animal incites a conspecific to change its position/location after approaching or agonistic interaction
	Nodding	Focal animal swings its head back and forth
	Lifting	Focal animal lifts another conspecific’s head or leg with its head/horns
	Staring	Focal animal is standing horn to horn in front of another conspecific with an uplifted head
	Pushing	Focal animal presses any part of its body against another conspecific making it change the position/location
	Chasing	Focal animal ‘follows’ another conspecific, which tries to keep the Focal animal at a distance, in a trotting manner
	Feigned attacking	Focal animal moves with a lowered head towards another conspecific and stops suddenly without causing body contact
	Attacking	Focal animal hits its horn against another conspecific
	Horn clashing	Escalated confrontation following ‘Attacking’ involving both animals hitting their horns against each other
Defensive	Avoiding	Focal animal changes its position or location after being approached by a conspecific, agonistic interaction with or agonistic vocalization from it
	Escaping	Focal animal moves away from a conspecific in a trotting manner after an agonistic interaction

### Receptive and non-receptive period

The receptive period was indicated by the male’s display of sexual behaviour (head placing, mounting or copulation) towards a specific female (Patton *et al.*, 1999; Swaisgood *et al.*, 2006). During the observations, the display of sexual behaviour was noted irrespective of the current focal animal. During observations, the receptive period lasted as long as sexual behaviour was observed on consecutive days, irrespective of the frequency of its occurrence. Non-receptive periods were defined as 3 and 6 days (±1) before the day of first display of sexual behaviour as well as 3 and 6 days (±1) after the last day of sexual behaviour display. If there were two receptive females present in the group, the receptive period was identified for each male–female dyad separately.

### Faecal sample collection and determination of androgen metabolite concentrations

Individual faecal samples were collected once to seven times a week over a period of 4 weeks on average, resulting in 19.5 ± 10.59 samples per study male (Supplementary 1). Faecal samples were collected in the morning after the study males were housed alone at night in order to ensure a clear assignment and to collect the samples at an approximately same time of the day (Brown *et al.*, 2001; Carlstead and Brown, 2005). Only in Zoo Schwerin we collected the faecal samples immediately after observation of defaecation, as the animals were not separated during the night. Consequently, the time lag between defaecation and sample collection did not exceed 12 h. Immediately after collection, samples were frozen and stored at −20°C until further analysis.

The quantification of fTM was based on an in-house testosterone assay and previously validated by Kretzschmar *et al.* (2004) for fTM determination in WR. This protocol was adhered to in the present study. Briefly, the respective antibody (provided by Professor Meyer, Weihenstephan, Germany) had been raised in rabbits immunized against 17α-OH-testosterone-HS-BSA. Cross-reactivities and HPLC immunograms are described in detail in Kretzschmar *et al.* (2004). Testosterone-3-CMO-peroxidase was used as the enzyme conjugate.

From each faecal sample, a representative subsample of 0.5 g was weighed after defrosting the sample. The subsequent steroid extraction procedure was conducted according to Kretzschmar *et al.* (2004). A total of 4.5 ml 90% methanol (MeOH) was added, followed by 30 min of shaking. The extract was centrifuged for 15 min at 1000 × g (Rotanta 46RC, Hettich GmbH & Co., Tuttlingen, Germany). An aliquot of 0.5 ml supernatant was then transferred to an Eppendorf vial and diluted 1:1 with distilled water. From this extract, 20 μl in duplicate were used for EIA analysis where it was combined with 100 μl of enzyme label (1:4000) and 100 μl of antibody (1:200 000).

The inter-assay coefficients of variation (CVs) (six assays), based on a low quality control sample and high quality control sample, both fitting into the linear range of the curve, were 11.35 and 11.30%, respectively. The intra-assay CVs, determined on two biological samples including low and high concentration (16 repeats, each in duplicate), were 11.90 and 4.37%, respectively.

The range of the calibration curve (testosterone) was 0.2–100 pg/20 μl. The linear range between B80 and B20 and ran between 1.04 and 17.40 pg/20 μl. All EIA measurements were performed in duplicate with acceptance criteria of a CV below 5%.

The fTM values were dated according to the delay of 1 day between the plasma testosterone concentrations and testosterone metabolite concentrations in faeces previously established by Kretzschmar *et al.* (2004).

### Statistical analysis

Statistical tests were calculated in ‘RStudio’ (version 3.5.5: RStudioTeam, 2016). The significance level was set at *P* ≤ 0.05; *P* < 0.1 was considered a statistical trend. Normal distribution of individual fTM levels as well as of mean fTM levels was verified using the Kolmogorov–Smirnov test (‘ks.test’ function) and Q-Q plots. Residuals were calculated for all linear mixed models (LMs) and linear mixed effect models (LMEs) in the study (‘resid’ function) and subsequently verified for normality as well as for homogeneity of variances.

### Correlation of age and fTM levels

In order to quantify the age-dependent development of fTM levels, a Pearson correlation between age and mean fTM levels was performed using the ‘cor.test’ function. For a further comparison between age groups an unpaired *t*-test (‘t.test’ function) was performed between sexually mature (*N*_SM_ = 12) and sexually non-mature (*N*_SNM_ = 4) study males.

### Effect of reproductive success and receptive females on fTM levels

To determine if reproductive success or presence of receptive females had an effect on fTM levels in sexually mature bulls (*N*_SM_ = 12), we calculated a LM (‘lm’ function) using mean fTM values as response variable and the interaction between the existence of sired offspring (yes/no) and availability of at least limited contact with receptive females (yes/no) as predictor factor (‘sired offspring*receptive females’). The best fitting model (final model) was determined via backward stepwise elimination procedure (‘car’ package, ‘Anova’ function; Zuur *et al.*, 2009). In order to further investigate the effects of significant main factors, comparisons between the factorial groups were conducted (‘lsmeans’ package, ‘lsmeans’ function). In the results section, only final models were reported.

### Differences across receptive and non-receptive periods

Differences in fTM levels as well as behaviour and vocalization during receptive and non-receptive periods were investigated in sexually mature males that had direct contact with receptive females on a daily basis (*N*_DC_ = 5, *N*_Dyads_ = 8).

In order to quantify behaviour and vocalization, daily proximity, olfactory, interaction as well as call rates were calculated for each focal male. Daily proximity rate was calculated for each one of the focal male-receptive female dyads by dividing the duration the focal male spent in close proximity to the respective female by the total observation time of the focal male on that day. Daily proximity rate was indicated in minutes per hour, thereby ranging from 0 to 60 min. Thus, a value of 60 indicated that the male–female dyad spent the full hour together, whereas a value of 0 indicated that the male–female dyad spent no time together. In doing so, we assessed the level of social cohesion between focal male and receptive female. Daily interaction rates were calculated by dividing the number of (i) affiliative, (ii) aggressive and (iii) defensive interactions of the focal male with the receptive female by the total observation time of the focal male on that day. Daily olfactory rates (‘marking, sniffing/flehming’) were calculated by dividing the number of displayed behaviour by the total observation time of the focal male on that day. Daily directed call rates were calculated for each call type by dividing the number of calls the focal male uttered to the receptive female by the total duration the focal animal spent in close proximity to the female on each observation day. Daily interaction, olfactory and call rates were indicated as number per hour.

For each one of the sexually mature males with direct contact to receptive females, daily fTM concentrations as well as daily behavioural rates and call rates during the receptive period (‘d0’) and non-receptive periods (‘d0±3’ and ‘d0±6’) of the respective female were determined. Mean values were calculated for each period including *±*1 day.

Subsequently, we investigated whether the different periods (‘d0–6’, ‘d0–3’, ‘d0’, ‘d0 + 3’, ‘d0 + 6’) had an effect on the mean values of daily fTM concentrations and behavioural rates by calculating LMEs (‘nlme’ package, ‘lme’ function) using the mean values as response variable and the periods as predictor variable, while controlling for ‘individual’ and ‘zoo’ as random factors. If there was a significant effect, a comparison among periods was conducted (‘lsmeans’ package, ‘lsmeans’ function, ‘Tukey’ adjustment for multiple comparisons). In the results section, only final models and significant pairwise comparisons between the receptive period (‘d0’) and the non-receptive periods (‘d0 ± 3’, ‘d0 ± 6’) are reported.

As both Pant and Hiss calls were only rarely observed, a statistical analysis was limited by zero inflation. Thus, we compared the number of dyads that uttered a call during the different periods (‘d0–6’, ‘d0–3’, ‘d0’, ‘d0 + 3’, ‘d0 + 6’) using a chi-square test calculated in ‘SPSS’ (IBM Corp., 2019).

## Results

### fTM levels

#### Correlation of age and fTM levels

**Figure 1:**
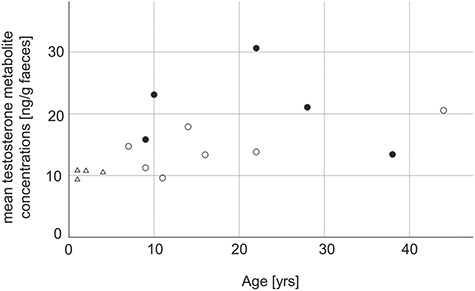
Mean fTM concentrations of study males Triangles = sexually non-mature males, blank circles = sexually mature males with no contact to receptive females, filled circles = sexually mature males with contact to receptive females.

The age of the study males showed a statistical trend to correlate positively with mean fTM levels (*r* = 0.478, *P* = 0.061, *N*_total_ = 16). When plotting the correlation, the two oldest study males (Floris, 38 years; Jambo, 44 years) stood out with comparatively low fTM levels (Fig. 1). A subsequent exclusion of these two individuals and a recalculation of the Pearson-correlation revealed that the age of the study males significantly correlated with the fTM levels (*r* = 0.640, *P* = 0.014, *N*_0–35_ = 14). An unpaired *t*-test revealed a significant difference with sexually mature bulls (≥5 years, *N*_SM_ = 12) having higher fTM values than non-mature ones (*N*_SNM_ = 4, *t* = 3.890, *P* = 0.002).

#### Effect of reproductive success and receptive females on fTM levels

The final model revealed that reproductive success did not have any effect on the fTM levels of sexually mature males (*N*_SM_ = 12, *t* = −1.116, *P* = 0.293), whereas presence of receptive females showed a statistical trend (*t* = −2.145, *P* = 0.060), suggesting that sexually mature males with access to receptive females (*N*_DC_ = 5) had higher fTM levels than the ones without any contact (*N*_NC_ = 7). When excluding the two oldest study males (Floris, Jambo) and recalculating the LM, reproductive success remained without a significant effect on fTM levels (*N*_SM_ = 10, *t* = −1.370, *P* = 0.213). However, the contact with receptive females turned out to be statistically significant, demonstrating that sexually mature study males with direct contact to receptive females (*N*_DC_ = 4) had higher mean fTM values than the ones without (*N*_NC_ = 6, *t* = −3.388, *P* = 0.012).

#### Differences in fTM levels across receptive and non-receptive periods

There was no significant effect of receptive and non-receptive periods on mean fTM values in the study males (*P* = 0.294).

### Behaviour and vocalization

#### Differences in behaviour across receptive and non-receptive periods

**Figure 2:**
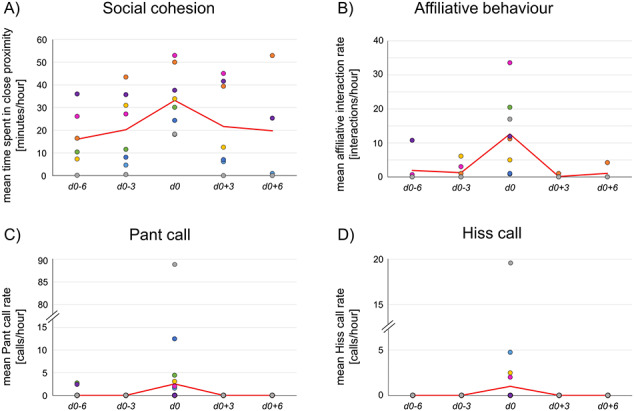
Mean values of (**A**) time spent in close proximity to receptive female, (**B**) affiliative interaction rate with receptive female, (**C**) Pant call rate towards receptive female and (**D**) Hiss call rate towards receptive female Each data point represents a male–female dyad during receptive (d0) and non-receptive (d0 ± 3/6) periods. Red coloured lines represent the mean values (A + B) or median values (C + D) over all dyads.

The period of female receptivity proved to have a significant effect on social cohesion (*P* < 0.001). Males spent significantly more time in close proximity to females during their receptive period compared to non-receptive periods 6 and 3 days before (*t* ≤ −3.401, *P* ≤ 0.020) as well as the non-receptive period 3 days after the receptive period (*t* = 3.076, *P* = 0.041; Fig. 2A, Table 3). A statistical trend further indicated that also during the non-receptive period 6 days after, males spend less time in close proximity to the females compared to their receptive period (*t* = 2.751, *P* = 0.080; Fig. 2A, Table 3).

**Table 3:** Mean values ± standard deviation for fTM concentrations (ng/g faeces), time spent in close proximity (social cohesion; min/hour) and behavioural rates (interactions/hour) as well as median values and range for call rates (calls/hour) in male adult SWR during receptive (d0) and non-receptive (d0 ± 3/6) periods

	d0–6	d0–3	d0	d0 + 3	d0 + 6
	*N_Dyads_ = 8*	*N_Dyads_ = 7*	*N_Dyads_ = 8*	*N_Dyads_ = 7*	*N_Dyads_ = 6*
fTM	18.84 ± 6.83	20.90 ± 6.31	20.10 ± 8.99	20.39 ± 6.24	16.30 ± 2.87
	*N_Dyads_ = 6*	*N_Dyads_ = 8*	*N_Dyads_ = 8*	*N_Dyads_ = 7*	*N_Dyads_ = 4*
Social cohesion	16.06 ± 13.13	20.24 ± 16.02	33.16 ± 13.25	21.65 ± 19.43	19.79 ± 25.00
Affiliative interactions	1.90 ± 4.34	1.26 ± 2.21	12.60 ± 11.03	0.14 ± 0.38	1.05 ± 2.10
Aggressive interactions	2.34 ± 2.29	0.00 ± 0.00	1.19 ± 1.77	1.38 ± 1.61	0.77 ± 1.00
Defensive interactions	2.35 ± 3.17	3.77 ± 3.87	4.20 ± 3.35	2.77 ± 3.67	1.62 ± 1.45
Marking	2.33 ± 3.06	5.02 ± 9.47	4.76 ± 7.61	3.45 ± 5.68	5.80 ± 11.59
Sniffing/Flehming	3.86 ± 3.44	3.93 ± 4.21	5.18 ± 3.59	2.66 ± 4.59	2.43 ± 2.21
Pant rate	0.00, 0.00–2.73	0.00, 0.00–0.00	2.53, 0.00–88.13	0.00, 0.00–0.00	0.00, 0.00–0.00
Hiss rate	0.00, 0.00–0.00	0.00, 0.00–0.00	1.00, 0.00–19.39	0.00, 0.00–0.00	0.00, 0.00–0.00

The period of female receptivity proved to have a significant effect on affiliative interaction rate as well (*P* < 0.001). The subsequent comparison showed that during the receptive period study males displayed more affiliative interactions towards females than during non-receptive periods before (*t* ≤ −3.330, *P* ≤ 0.024) and after (*t* ≥ 3.170, *P* = ≤ 0.034; *Fig*. 2B, Table 3). An effect of receptive and non-receptive periods on the aggressive interaction rate between study males and females was revealed (*P* = 0.017). However, the pairwise comparison did not show any significant difference between the receptive period and either one of the non-receptive periods before or after. For the defensive interaction rate, no significant effect of the period was found (*P* = 0.508).

Neither marking nor sniffing/flehming behaviour turned out to be significantly affected by the period in the study males (*P* > 0.506).

#### Differences in vocalization across receptive and non-receptive periods

Both call types were found to be uttered by significantly more males during the females’ receptive period than during their non-receptive period (Pant: χ^2^ = 17.572, df = 4, *P* < 0.001; Hiss: χ^2^ = 14.224, df = 4, *P* = 0.007). When plotting the mean dyadic call rates, both calls clearly peaked during the receptive period with six out of eight male-receptive female dyads uttering Pant calls (*N*_d0_ = 8, median_d0_ = 2.53 calls/hour, range_d0_ = 0.00–88.13 calls/hour; Fig. 2C, Table 3) and four out of eight uttering Hiss calls (*N*_d0_ = 8, median_d0_ = 1.00 calls/hour, range_d0_ = 0.00–19.39 calls/hour; Fig. 2D, Table 3).

## Discussion

The findings of the present study demonstrate an age-dependent increase in fTM concentrations and indicate an underlying female effect enhancing the androgen levels in sexually mature SWR males. At the same time, constant fTM levels throughout the receptive and non-receptive periods of females suggest that androgen concentrations may not be the main driver of behavioural changes during courtship and mating.

The positive correlation of fTM concentrations and age illustrated a gradual increase in androgen levels from juvenile age up until adulthood in WR males. This rise seemed to be followed by a decline in the advanced age, as two of the oldest study males that were more than thrice as old as the average displayed comparatively lower mean fTM values. Similar age-associated developments of androgen concentrations in males have been characterized in numerous studies on other mammalian species, which also describe a rise in testosterone levels from infancy to puberty (e.g. Rota *et al.*, 2002; Behringer *et al.*, 2014; Edwards *et al.*, 2015) and an age-related decline throughout senescence (e.g. Machida *et al.*, 1981; Ellison *et al.*, 2002; Altmann *et al.*, 2010; Nelson, 2011; Thompson *et al.*, 2012). The significant difference that revealed higher fTM levels in sexually mature compared to sexually non-mature bulls additionally supports the age-dependent rise in androgen levels, emphasizing once again the importance of androgens for sexual maturation. Our findings are in line with previous comparisons between adult and subadult or juvenile WR males conducted by Kretzschmar *et al.* (2004) and Ververs (2018) and, moreover, provide first insights into the development of androgen concentrations in different age stages of male WR. Nevertheless, future studies would require a larger sample sizes in all age categories, particularly to confirm fTM decline in older bulls and generally enable a thorough investigation of the initial insights into the age-related development of fTM levels.

The investigation of further factors potentially affecting androgen levels in sexually mature bulls in captivity showed that previous reproductive success does not have any influence. Bulls that had successfully sired offspring before did not differ from bulls without offspring with regard to mean fTM levels. While these results match the findings of Brown *et al.* (2001), who also did not find any difference between proven and unproven males, they contradict the outcome of Edwards *et al.* (2015), who found that in black rhinoceroses (BR) proven bulls have significantly higher faecal androgen metabolite levels than unproven ones. This contrast might be attributed to the different social organization of the two African rhinoceros species: adult WR males are described as solitary, only associating with females when they are approaching oestrus, as well as territorial, occasionally accepting subordinate satellite bulls (Owen-Smith, 1971, 1973; Pienaar, 1994). Conversely, BR males are characterized as less strictly territorial, living in loose spatially scattered clans with neighbouring bulls, maintaining a clear hierarchical structure (Penny, 1987). Receptive females entering the BR males’ territory might therefore be mounted by several bulls successively according to their ranks, which poses a classical setting for sperm competition (Penny, 1987). Androgen levels are not only closely associated with spermatogenesis (Heldmaier *et al.*, 2013) but also with aggressive behaviour and, therefore, dominance and ranks within group hierarchies in many species (e.g. Beehner *et al.*, 2006; Muehlenbein and Watts, 2010; Nelson, 2011; Wang *et al.*, 2012). Thus, it could be assumed that high fTM levels might be directly related to access to receptive females and ultimately to reproductive success in BR males. In WR, the competitive pressure might not be as direct and ubiquitous, as there is usually only one bull courting a receptive female for up to several weeks in his own territory, seldom encountering other males (Owen-Smith, 1971, 1973, 1988). Hence, higher androgen levels might not to be directly equated with previous breeding success in WR males as they are in BR males.

In contrast, the presence of receptive females proved to have an effect on the fTM levels. Bulls that were kept together with regularly cyclic females possessed higher faecal androgen levels than the ones that were kept with non-cyclic females. This female effect has been previously described by Christensen *et al.* (2009) as well as Kretzschmar *et al.* (2004). Our study not only supports the previous findings but also suggests that it is not the mere presence of females that affects the androgen states of WR bulls but rather their regular oestrus cycle, which triggers the increase in fTM levels. This assumption might also provide an explanation as to why Ververs (2018) did not find any differences in faecal androgen levels between isolated males and the ones housed in mixed-sex groups. For his analysis, he only took one-time samples for each study male and did not consider the cycle state of the females. Hence, his study did not account for the possible presence of non-cyclic females in the groups and focused on a series of single events rather than a continued period of time.

When taking a closer look at the development of fTM levels throughout the females’ cycle phases, we found constant androgen metabolite concentrations during receptive as well as non-receptive periods. In contrast, there is some evidence from other mammalian species indicating the possibility of a peak in androgen levels and accordingly a rise in sexual activity coinciding with the ovulation of the female (Yagil and Etzion, 1980; Bonney *et al.*, 1982; Kleiman, 1983), suggesting testosterone to be a potential determining driver of socio-sexual behaviour in males. We could not confirm that for WR males, there being two possible explanations for this. While the previously mentioned studies were conducted in seasonal breeders that greatly rely on synchronized reproductive behaviour, WR are mainly described as non-seasonal breeders that are able to mate throughout the year even if there is a preference for some seasons (Kretzschmar *et al.*, 2004; Ververs *et al.*, 2017). Therefore, we would not suspect a particular peak in sexual activity or in corresponding androgen levels. Secondly, the unchanging androgen levels might be an effect of captivity, as the receptive females are immediately accessible and so males need to be prepared for mating almost continuously, given that the regular cycle length in WR is one, in some cases 2 months (Schwarzenberger *et al.*, 1998; Patton *et al.*, 1999; Brown *et al.*, 2001; van der Goot *et al.*, 2015b). Hence, we would expect males with direct contact to regular cyclic females to have generally elevated fTM levels, which indeed matches our mentioned comparison between bulls that were kept around females with a regular cycle and the ones that were kept with non-cyclic females. So far, our results on the development of androgen metabolite levels in males throughout the cycle period are the only available data for WR. However, field studies would be necessary to clarify whether these findings can in fact be explained by the non-seasonality of breeding in this species or if it is rather a consequence of captivity and the permanent availability of receptive females.

While the fTM levels of the males did not change during the females’ receptive and non-receptive periods, we could still detect a significant rise in social cohesion as well as affiliative interactions with the respective females during their receptive period. The peak in affiliative interactions might not be surprising, as we defined the receptive period of females by means of sexual behaviour displayed towards them, which is composed of male affiliative behaviour. Moreover, sexual behaviour implies being in close proximity by definition, therefore entails increased social cohesion. However, the definition of the receptive period took only the mere presence or absence of sexual behaviour into account, irrespective of the frequency of occurrence. In contrast, both affiliative interactions and social cohesion were presented as rates per hour, thereby representing the qualitative dimensions that significantly peaked during the receptive period. These findings emphasize the natural behaviour of WR males comprising guarding and accompanying the female during oestrus, therefore spending more time in close proximity as well as displaying more socio-positive behaviour, including following, snout and body contact, ano-genital sniffing, head placing and mounting (Owen-Smith, 1973, 1975; Penny, 1987). A larger sample size, especially regarding the non-receptive periods, could lend the present results a larger statistical power, which is admittedly limited in cases where data on only few dyads was available. Nevertheless, our results confirm that WR males in captivity display the same socio-sexual behavioural repertoire as their wild counterparts. At the same time, this implies that androgens are not necessarily the main driver of behavioural changes in captive bulls during courtship and mating, but they are rather mediated by other factors instead. Here, one of the potential parameters could be the olfactory indicators that the bulls perceive when they sniff or flehm. Previous studies clearly demonstrated the essentiality of olfactory communication for WR with regard to intra-specific recognition, territory maintenance as well as determination of receptive females (Marneweck *et al.*, 2017, 2018). Detecting the female’s cyclic state by absorbing olfactory cues from dung or urine might therefore be an important triggering factor for the males to adjust their behaviour. However, we could not find an expected difference in olfactory behaviour that coincided with the peaks in social cohesion or affiliative behaviour. This, in turn, implies that olfactory communication is fundamentally essential independent of receptive or non-receptive periods and it is the qualitative information the males perceive rather than the olfactory behavioural rate that determines the changes in their socio-sexual behaviour.

Even though we would have expected an effect of receptive period on the rates of aggressive and defensive interactions with females, we could not find any difference in agonistic behaviour between receptive and non-receptive phases. A rise in aggression during the mating period has been previously described in various other mammals (e.g. Cavigelli and Pereira, 2000; Gould and Ziegler, 2007). However, the majority of these elevated behavioural rates included male–male interactions described more in a mate competition context, which was not applicable in the present study, as there was always only one sexually mature bull available in a group. Hence, in order to complete the picture of behavioural changes related to reproduction in male WR, further studies in the field enabling the observation of natural social group compositions would be needed.

Moreover, our results emphasize the role of vocal communication for courtship and mating in WR. Despite the overall rare display of Pant and Hiss calls, we were still able to show that most of the study males uttered both call types almost exclusively during the females’ receptive period, when the call rates distinctly peaked, too. This increase in call rates matches those studies on vocalization in other mammals, which demonstrated elevated call rates associated with courtship and mating behaviour (Buesching *et al.*, 1998; Wielebnowski and Brown, 1998; Lindburg *et al.*, 2001; Leong *et al.*, 2003). Furthermore, the peculiarity of the Pant call, reportedly showing the male-typical variation Hic in the wild (Owen-Smith, 1973; Cinková and Policht, 2016; Cinková and Shrader, 2020), became evident, as calls that would have corresponded to the Hic description of Cinková and Shrader (2020) were only recorded in two of the study bulls. These findings suggest that the differences between the two call variations Pant and Hic might be more pronounced in the wild, where the territorial males have neighbouring competitors and are required to establish and maintain their home ranges while guarding receptive females. Hence, due to lack of competition and the rather predictable zoo environment, there might less need for two different call types in captivity than in the wild.

The present results not only lend support to the previous findings on the androgen metabolite concentrations and the factors affecting them in WR males but they also present first insights on the development over different age classes as well as during the females’ cycle phases. Taken together, we can conclude that while the previous reproductive success might not be sufficiently decisive to predict androgen metabolite concentrations, the presence of cyclic females does enhance fTM levels in WR males. Putting these findings into the scope of application for captivity management would mean that translocating a successfully breeding bull into another group might not inevitably lead to a continuously high reproductive rate, as it greatly depends on the females’ reproductive state. However, this does not exclude the possibility of the introduction of a new bull stimulating a female’s cycle, as it has been previously reported for a few cases (e.g. Patton *et al.*, 1999). A more systematic approach involving a higher sample size and specifically focusing on the effects of the introduction of new mating partners would be needed for a more coherent picture.

Conclusively, we determined male behavioural as well as vocal indicators that coincide with the females’ receptive period and comply with the respective repertoire in the field. These particular findings create an applicable basis for non-invasive monitoring of reproductive behaviour of WR in captivity. The display of significantly increased affiliative behaviour rates and close proximity as well as higher call rates of the contact call Pant and agonistic call Hiss in males clearly signals imminent oestrus.

## Funding

This work was supported by the Serengeti-Park Stiftung and the Deutsche Forschungsgemeinschaft (SCHE 1927/2-1).

## Author contributions statement

JJ collected part of the data, performed part of the video analysis, analysed the data, wrote the manuscript and prepared all figures. JW and MD supervised the endocrine analysis. MS provided the grant, designed and supervised the study and contributed to the data analysis. All authors reviewed the manuscript.

## Data availability statement

Background data used for the manuscript are included in the supplementary information. Video and audio data are stored at the Institute of Zoology, University of Veterinary Medicine Hannover, Germany, and are available on reasonable request.

## Supplementary Material

Supplementary_Information_Jenikejew_et_al_2021_coab026
