# Supplementary material for: The female effect—how female receptivity influences faecal testosterone metabolite levels, socio-positive behaviour and vocalization in male Southern white rhinoceroses
Source: Conserv Physiol. 2021 Apr 28;9(1):coab026. doi: 10.1093/conphys/coab026 (PMC8084027; doi:10.1093/conphys/coab026)
Supplement: Supplementary_Information_Jenikejew_et_al_2021_coab026 [file supplementary_information_jenikejew_et_al_2021_coab026.docx]

# Supplementary Information

**Supplementary 1:** Number of collected faecal samples and mean fTM concentrations [ng/g faeces] in study males.

| **ID** | **Zoo** | **Number of faecal samples** | **Mean fTM concentrations**  **[ng/g faeces]** |
| --- | --- | --- | --- |
| **Floris** | Osnabrück | 30 | 13.43 |
| **Bantu** | Augsburg | 45 | 15.70 |
| **Amari** | Dortmund | 27 | 11.26 |
| **Dino** | Erfurt | 30 | 13.84 |
| **Martin** | Hodenhagen | 16 | 30.64 |
| **Abasi** | Hodenhagen | 16 | 10.51 |
| **Dinari** | Hodenhagen | 6 | 10.75 |
| **Lekuru** | Gelsenkirchen | 19 | 9.63 |
| **Kimba** | Schwerin | 12 | 23.11 |
| **Harry** | Münster | 26 | 21.06 |
| **Amiri** | Münster | 7 | 10.82 |
| **Benny** | Amnéville | 22 | 17.92 |
| **Timbo** | Amnéville | 6 | 9.35 |
| **Shaka** | Knuthenborg | 24 | 13.36 |
| **Jambo** | Planéte Sauvage 1 | 13 | 20.55 |
| **Goliath** | Planéte Sauvage 2 | 13 | 14.75 |
